# What is the extent and quality of documentation and reporting of fidelity to implementation strategies: a scoping review

**DOI:** 10.1186/s13012-015-0320-3

**Published:** 2015-09-07

**Authors:** Susan E. Slaughter, Jennifer N. Hill, Erna Snelgrove-Clarke

**Affiliations:** Faculty of Nursing, University of Alberta, Edmonton, Alberta Canada; Department of Veteran’s Affairs, Spinal Cord Injury Quality Enhancement Research Initiative, Hines, IL USA; School of Nursing, Department Obstetrics/Gynecology, Dalhousie University, Halifax, Nova Scotia Canada

## Abstract

**Background:**

Implementation fidelity is critical to the internal and external validity of implementation research. Much of what is written about implementation fidelity addresses fidelity of *evidence-informed interventions* rather than fidelity of *implementation strategies*. The documentation and reporting of fidelity to implementation strategies requires attention. Therefore, in this scoping review, we identify the extent and quality of documentation and reporting of fidelity of implementation strategies that were used to implement evidence-informed interventions.

**Methods:**

A six-stage methodological framework for scoping studies guided our work. Studies were identified from the outputs of the Effective Practice and Organization of Care (EPOC) review group within the Cochrane Database of Systematic Reviews. EPOC’s primary focus, implementation strategies influencing provider behavior change, optimized our ability to identify articles for inclusion. We organized the retrieved articles from the systematic reviews by journal and selected the three journals with the largest number of retrieved articles. Using a data extraction tool, we organized retrieved article data from these three journals. In addition, we summarized implementation strategies using the EPOC categories. Data extraction pertaining to the quality of reporting the fidelity of implementation strategies was facilitated with an “Implementation Strategy Fidelity Checklist” based on definitions adapted from Dusenbury et al. We conducted inter-rater reliability checks for all of the independently scored articles. Using linear regression, we assessed the fidelity scores in relation to the publication year.

**Results:**

Seventy-two implementation articles were included in the final analysis. Researchers reported neither fidelity definitions nor conceptual frameworks for fidelity in any articles. The most frequently employed implementation strategies included distribution of education materials (*n* = 35), audit and feedback (*n* = 32), and educational meetings (*n* = 25). Fidelity of implementation strategies was documented in 51 (71 %) articles. Inter-rater reliability coefficients of the independent reviews for each component of fidelity were as follows: adherence = 0.85, dose = 0.89, and participant responsiveness = 0.96. The mean fidelity score was 2.6 (SD = 2.25). We noted a statistically significant decline in fidelity scores over time.

**Conclusions:**

In addition to identifying the under-reporting of fidelity of implementation strategies in the health literature, we developed and tested a simple checklist to assess the reporting of fidelity of implementation strategies. More research is indicated to assess the definitions and scoring schema of this checklist. Careful reporting of details about fidelity of implementation strategies will make an important contribution to implementation science.

**Electronic supplementary material:**

The online version of this article (doi:10.1186/s13012-015-0320-3) contains supplementary material, which is available to authorized users.

## Background

Implementation fidelity is generally defined as the degree to which a program is implemented as it was intended in the original program model or protocol; however, definitions vary across disciplines making shared understanding of approaches and findings difficult [1–4]. It is recognized as a key component to evaluating evidence-informed interventions [5] at the “implementer-level” [3] such as clinical practice guidelines [4] and to evaluating implementation strategies [6] at the “programmatic-level” [3] such as educational or financial support [6]. The importance in the distinction of these terms is discussed later. Implementation fidelity is critical to the internal and external validity of implementation research. Without it, accurate conclusions about an intervention cannot be drawn as unknown factors may have influenced the outcome(s).

Health behavior change researchers, in collaboration with the National Institutes of Health, developed a comprehensive approach to the fidelity of health behavior change interventions [7]. This Behavior Change Consortium (BCC) recommended five categories of treatment fidelity strategies with the first three categories (study design, provider training, and treatment delivery) focusing on the provider and the last two categories (receipt of treatment and enactment of treatment skills) focusing on the patient.

At the other end of the spectrum, there are measures that offer a more flexible approach to assessment of care provider behavior. Dusenbury et al. [1], adopted from Dane and Schneider [8], acknowledges that five loosely connected elements have been associated with a holistic picture of *implementation fidelity*: adherence, dose, program delivery, participant engagement, and program differentiation, noting that program differentiation never seemed to be measured [1]. Elliott et al. argued that the process of identifying core program elements for program differentiation has serious limitations [9]. Others have since cited these five elements [2, 4]; however, not everyone agrees that each of these components should be included in an evaluation of fidelity [5, 9]. Moreover, the health behavior change of the BCC focuses on fidelity of the treatment aligned with diverse patient populations, while Dusenbury’s et al. [1] framework focuses on the fidelity of implementation strategies aligned with the care provider. Several other examples of assessing fidelity were also found including the following: (1) an approach which involved a simple, single-item subjective assessment, completed by implementers, comparing program delivery to the original implementation plan on a 4-point scale [10] and (2) a review of fidelity monitoring which used rating scales to assess adherence to study interventions [11].

In a critical review of conceptualizations of implementation fidelity, Carroll et al. [2] found that existing research focused on adherence with only a few studies measuring participant responsiveness and quality of delivery. Based on this review, they developed a Conceptual Framework for Implementation Fidelity [2] that has guided the work of others [12] and is recommended for monitoring implementation fidelity but focuses on the delivery of the evidence-based intervention or program with the implementation strategy as a moderating factor.

Establishing a distinction between the terms “programmatic level” or implementation strategy adherence and “implementer level” or evidence-informed clinical intervention adherence [3] is of particular interest given the growing attention to implementation research in health science communities traditionally focused on clinical effectiveness studies [4]. In addition, clarifying the distinction is relevant with the advent of studies, such as hybrid trial designs [13], that simultaneously assess the effects of an implementation strategy on provider behavior change (the use of a clinical intervention) and of a clinical intervention on patient outcomes or patient behavior change. Only a few studies have made this distinction [3, 4]. Examples of evidence-informed treatment interventions include cancer-screening processes [14], prescribing practices [15], and introducing cognitive behavior therapy [16] or obesity management [17] to primary care. Examples of cited implementation strategies include distribution of educational materials to intervention providers or to patients to influence provider behavior, audit and feedback, and reminders for providers [14].

There is a lack of conceptual clarity in what constitutes an implementation strategy [18, 19]; many implementation strategies may also be utilized as evidence-informed interventions. An evidence-based intervention can stand on its own (without an implementation strategy); however, an implementation strategy cannot exist without an evidence-based intervention because it supports implementation of that intervention. Taxonomies in the literature include the recently developed Expert Recommendations for Implementing Change (ERIC) taxonomy focused on identifying, developing, and testing implementation strategies and consists of an expanded list of 73 implementation strategies [18] and the Cochrane Effective Practice and Organisation of Care (EPOC) taxonomy which has published 100 systematic reviews of the literature, updated every 3 years, for each of the 47 implementation strategies included in the taxonomy [20]. These taxonomies often include patient-mediated interventions as well, which rely on actions of a patient to influence provider behavior such as direct advertising to patients about a drug by pharmaceutical companies which may result in increases in provider prescribing of that drug.

The literature on fidelity of implementation exists in a broad range of fields [5], all of which would benefit from a unified language. For example, terms used to describe “programmatic level” adherence, or the degree to which implementation strategies are utilized as designed [3], include but are not limited to the following: “fidelity of implementation” [1], “implementation fidelity” [2, 4], “implementation adherence” [3], “program adherence” [8], and “strategy fidelity” [6]. To illustrate, if guidelines for appropriate prescribing practices (intervention) were implemented using unit-level audit and feedback (implementation strategy) at monthly physicians meetings over the course of 6 months, adherence would be assessed by counting the number of sessions and when they occurred and how many physicians attended the meeting. Alternatively, the terms used to describe individual “implementer level” adherence, or the degree to which an implementer follows the intervention as specified [3], include but are not limited to the following: “fidelity monitoring” [11], “treatment fidelity” [7, 10], and “program fidelity” [5]. Using the example above, fidelity to guidelines for appropriate prescribing practices (intervention) would be assessed using adherence to the provider prescribing behaviors detailed in the guidelines. For the remainder of this article, we will refer to programmatic level adherence as “fidelity to an implementation strategy” and implementer level adherence as “fidelity to an evidence-informed intervention”.

A variety of elements can affect the delivery of an implementation strategy. These elements include, but are not limited to the following: the setting where an intervention is implemented [21], the health care providers targeted for the behavior change [22, 23], and the complexity of the implementation strategy [24]. Variations in quality of delivery and documentation of delivery can adversely affect the internal validity of the study [2] and can lead to difficulty in accounting for which component(s) of the strategy influenced the implementation outcome(s) and ultimately impact the generalizability of the findings related to the use of that implementation strategy.

The concept of fidelity is not a new one; however, measurement of fidelity is limited and there is no consensus on how to do it [4, 11]; we found several examples of systematic reviews measuring fidelity of the evidence-informed intervention. In a review of literature published between 1980 and 1994, Dane and Schneider found that only 39 (24 %) of the 162 studies featured specified procedures for documenting fidelity of which only 13 (8 %) considered variations in integrity as a potential influence on the effects of the program [8]. In 2005, Borelli et al. found only 27 % of the 342 studies in their review assessed whether the intervention was delivered as specified [25]. In 2011, Gearing et al. conducted a comprehensive review of 24 meta-analyses and review articles over a 30-year span and found large variations in reporting of aspects of fidelity [26]. In 2013, Schober, Sharpe, and Schmidt examined reports of strategies for maintaining fidelity and found that fidelity assessment reporting were “generally poor” and reported between 22 and 56 % of fidelity criteria [27].

Definitions and measurement of fidelity vary across the studies reporting these assessments. In a review of 133 studies on implementation fidelity in curriculum intervention research, O’Donnell summarized findings stating there are too few studies to inform measurement of fidelity of implementation and how extent of fidelity is related to study outcomes and called upon the field for “improvements in clarity, conceptualization, and measurement” [28]. Documenting and reporting the elements of an implementation strategy are necessary for an assessment of fidelity [19] and an understanding of the impact of the strategy on implementation across implementation settings, especially in cases where local context makes adaptations to the strategy necessary. Identifying a shared conceptual understanding of fidelity that spans fields of study offers the opportunity to move implementation science and fidelity measurement forward.

Attention to fidelity of implementation strategies has not been the publication standard. While approaches for scoring fidelity have been suggested [1, 2, 26], few researchers report systematic documentation of fidelity to implementation strategies much less apply these scoring methods. The purpose of this article is to assess the extent and quality of documentation of fidelity to implementation strategies by conducting a scoping review.

## Methods

Consistent with a scoping review, it was not our intent to draw conclusions about the findings of the studies: we neither rated the empirical quality nor conducted any sort of synthesis of study findings [29]; however, we did evaluate the extent and quality of documentation regarding fidelity. Also, consistent with a scoping review, we set out to rapidly examine or explore the nature of an existing identified literature, rather than conduct a synthesis of the wider literature [30].

We followed a 6-step methodological framework for scoping studies which was initially developed by Arksey and O’Malley [31] and further clarified and enhanced by Levac et al. [29]. The framework for a scoping study includes the following steps: (1) identifying the research question; (2) searching for relevant studies; (3) selecting studies; (4) charting the data; (5) collating, summarizing, and reporting the results; and 6) consulting stakeholders to inform or validate study findings.

### Step 1: identifying the research question

The research question guiding the review was “What is the extent and quality of documentation of fidelity to implementation strategies?”

### Step 2: searching for relevant studies

Our aim was to identify a sample of interventional study articles that utilized implementation strategies to target provider behavior change and patient outcomes; identifying knowledge translation articles has been noted as challenging by some authors [32, 33]. Specialized search filters have been developed to find knowledge translation articles in CINAHL and MEDLINE, but testing of these filters revealed a specificity of only 65 and 50 %, respectively [32, 33]. Due to the complexity of searching for fidelity and knowledge translation articles, and the potential for retrieval of a large number of irrelevant studies if we conducted a traditional search using a large set of terms in multiple databases, we chose to use an extremely focused search. Our goal was to explore the concept of fidelity of implementation strategies from a smaller set of articles that likely reported this; therefore, in the fall of 2013, we adopted an alternative search strategy which involved accessing the 47 systematic reviews listed under the Effective Practice/Health Systems topic in the Cochrane Database of Systematic Reviews [20] and the articles cited in each systematic review. These systematic reviews are intended to explore each of the 47 implementation strategies in the EPOC Taxonomy and are updated regularly by the EPOC group.

### Step 3: initial selection of studies

Using the reference lists in each of the systematic reviews, we identified the articles included in each review, which resulted in large number of articles. To facilitate feasibility, we utilized a strategy similar to that of Kanakamedala and colleagues [34], in which we categorized the articles by the journals of publication and identified the journals in which 30 or more articles were published. This resulted in three high-impact leading medical journals and their articles. To summarize, we included articles that identified implementation strategies intending to change health care provider change, articles reporting at least one type of implementation strategy, and articles with study designs that were either randomized controlled trials or cluster randomized controlled trials.

### Step 4: extracting the data

The data extraction tool was developed with initial consensus by all authors; following its development, it was piloted with an initial set of articles (~30 %), utilized for independent reviews by the author pairs, and discussed in consensus meetings between author pairs. As a result of this pilot, the authors subsequently devised a set of definitions for populating each column (Additional file 1).

The three authors (SES, JNH, and ESC) conducted independent reviews (two authors per article) using the data extraction table; we excluded a small number of articles from the review because they did not include health care provider behavior change. Author pairs met to discuss their independent reviews and achieve consensus regarding the details to include in the final table; we did not seek referenced companion articles for additional details about the studies.

After the initial collation of information from the articles was complete, the authors focused on a second level of analysis of the category “fidelity to the implementation strategy”. We adapted a scoring schema from Dusenbury et al. [1] to assess the quality of fidelity documentation in relation to adherence, dose, and participant response on a 3-point scale (0–2) (see Table 1). We opted not to assess two of Dusenbury’s elements of fidelity (quality of program delivery and program differentiation) because these components focused on the characteristics of the program or *evidence-based intervention* to be implemented rather than on fidelity. Others have agreed with this assessment [5]. Definitions for each element and the scoring schema went through several revisions before settling on final definitions and scoring criteria which formed a checklist (Table 1). Each included study has a single fidelity score, even when several implementation strategies were utilized in a particular study. The scoring schema outlined in Table 1 clarifies how this is possible for each of the three domains. A score of 2 suggests that all implementation strategies met the condition. A score of 1 suggests that some but not all of the implementation strategies met the condition. A score of 0 suggests that at least one condition was not met.

**Table 1:** Scoring rubric for documentation and recording of fidelity to the implementation strategy

Domain	Dusenbury^a,b^ definition	Adapted operational definition	Checklist questions
Adherence	The extent to which implementation of particular activities and methods is consistent with the way the program is written	Specifying the implementation strategy(s) *and* evidence of the extent to which this/these implementation strategy(s) took place	To score 2, both conditions must be present/yes
			Condition 1: Does the study describe *all* implementation strategies used? AND
			Condition 2: Does the study provide detail on how *all* implementation strategies were carried out?
			To score 1, both conditions must be present/yes
			Condition 1: Does the study describe *some but not all* implementation strategies used? AND
			Condition 2: Does the study provide detail on how *some but not all* implementation strategies were carried out?
			To score 0, one condition may be present, OR no conditions may be present/yes:
			Condition 1: Does the study *describe all* or *some* implementation strategies used? OR
			Condition 2: Does the study *provide detail on how all* or *some* of the implementation strategies were carried out?
Dose	The amount of the program content received by participants	Proportion of intervention providers who received the implementation strategy(s) (i.e., number of people *and* specific strategy received)	To score 2, both conditions must be present/yes:
			Condition 1: Does the study provide a description of the number of people receiving *all* of the implementation strategies? AND
			Condition 2: Does the study provide a description of the strategy or strategies *all* of the groups received?
			To score 1, both conditions must be present/yes
			Condition 1: Does the study provide a description of the number of people receiving *some but not all* of the implementation strategies? AND
			Condition 2: Does the study provide a description of the strategy or strategies for *some but not all* of the groups?
			To score 0, one condition may be present, OR no conditions may be present/yes:
			Condition 1: Does the study provide a description of the number of people receiving *some* or *all* of the implementation strategies? OR
			Condition 2: Does the study provide a description of the strategy or strategies for *some* or *all* of the groups?
Participant Responsiveness	The extent to which participants are engaged by and involved in the activities and content of the program	Extent to which intervention providers are involved in the development of the implementation strategy, evaluation of the implementation strategy or their receptivity to the implementation strategy *and* extent of involvement	To score 2, both conditions must be present/yes:
			Condition 1: Does the study state participants involvement in the development, evaluation, or receptivity to the implementation strategy? AND
			Condition 2: Does the study provide a description of the *extent* of participant involvement in the development, evaluation, or receptivity to the implementation strategy?
			To score 1, *one* condition must be present/yes
			Condition 1: Does the study provide a description of the number of people receiving *some but not all* of the implementation strategies? AND
			Condition 2: Does the study provide a description of the strategy or strategies for *some but not all* of the groups?
			To score 0, *no* conditions are present/yes
			Condition 1: Does the study provide a description of the number of people receiving *some* or *all* of the implementation strategies? AND
			Condition 2: Does the study provide a description of the strategy or strategies for *some* or *all* of the groups?

^a^Dusenbury [2]

^b^We chose not to include the *Quality of Program Delivery* and *Program Differentiation* components as part of our scoring since they relate to the fidelity to the evidence-informed intervention

The scoring schema integrated both the extent and quality of fidelity. For example, adherence was defined as the extent to which the implementation of particular activities or methods was consistent with the way the program was written (quality). To illustrate, if the plan calls for the use of multiple implementation strategies (e.g., provider education and audit and feedback) to support guideline implementation (intervention) and includes two 1-hour sessions to be delivered 6 months apart (provider education) and weekly group feedback sessions based on performance data (audit and feedback), was this plan followed? Were deviances from the plan and the reason for the deviance documented? Dose was defined as the amount or extent to which individuals received all of the program content delivered. To continue with the example above, how many of the targeted staff attended all of the educational sessions provided? How many attended the feedback sessions? How many attended just one or none of the sessions? Participant response was defined as the extent to which participants were engaged and involved in the development of the implementation strategy, evaluation of the implementation strategy, or their receptivity to the implementation strategy. In the example above, were staff involved in developing the strategies (e.g., planning or content), providing direct feedback on the strategies (e.g., via survey or interviews), and/or was there indirect feedback gathered from the individual utilizing the strategies on provider receptivity?

Based on independent reviews using the new scoring schema, the author pairs met to discuss discrepancies and achieve consensus. An a priori criterion of 85 % inter-rater reliability was set for each component of the fidelity score. This was calculated using proportion agreement. Consensus was reached on all fidelity scores reported in the article.

### Step 5: collating, summarizing, and reporting the results

We collated the data entered into the data extraction table according to each category in the table; in some cases, we created subcategories to facilitate collation. For example, we organized the year of publication by decade, inductively developed categories for the various evidence-informed interventions, and organized the implementation strategies according to the EPOC implementation strategy categories [20]. We also noted examples of articles in which the documentation of fidelity to the implementation strategy was particularly well done.

### Step 6: consulting stakeholders

The origins of this scoping review evolved from a breakout discussion group at an annual Knowledge Utilization Colloquium in 2013 [35]. In addition to the early discussions with participants at the colloquium, and as delineated in the scoping review approach, we consulted with two stakeholder implementation experts by e-mail during the planning stage of the scoping review and with one additional stakeholder face-to-face prior to finalizing this report.

## Results

**Fig. 1:**
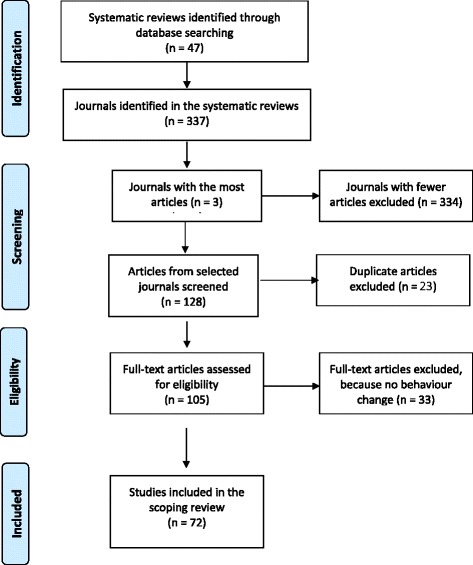
Search and selection process

From the references included in the 47 systematic reviews, we identified 1158 articles published in 337 journals. The three journals including the most articles were the British Medical Journal (*n* = 54), Journal of the American Medical Association (*n* = 40), and Medical Care (*n* = 34). Thus, the initial search yielded 128 articles. With duplicates removed, there were 105 articles remaining. After reviewing the full articles for the 105 articles, it became apparent that there was no health provider behavior change for 33 of these. Thus, 72 articles were selected for inclusion in this scoping review. Figure 1 summarizes the search and retrieval process.

### Summary of included articles

The 72 included articles were published between 1980 and 2011, with 18 articles published between 1980 and 1989, 26 published between 1990 and 1999, and 28 published between 2000 and 2011 (see Additional files 2 and 3). The 72 studies were conducted in 13 countries: the USA (*n* = 36), the UK (*n* = 16), Canada (*n* = 9), Australia (*n* = 3), the Netherlands (*n* = 3), Scotland (*n* = 3), India (*n* = 1), Norway (*n* = 1), Portugal (*n* = 1), Spain (*n* = 1), Sweden (*n* = 1), Wales (*n* = 1), and Zambia (*n* = 1). The majority of research designs in these studies were either randomized controlled trials (*n* = 37) or cluster randomized controlled trials (*n* = 15). Though we expected to find definitions for fidelity and a conceptual framework for fidelity in each article, none were found.

While the primary focus of this review was on fidelity to implementation strategies, some examples of the evidence-informed interventions and the related implementation strategies targeting these interventions are provided. In one example of the many pharmacologically focused interventions, the use of antibiotics for uncomplicated bronchitis was targeted using four implementation strategies: mass mailings of education materials to the public (patient-mediated intervention), the distribution of educational materials to patients and clinicians, educational meetings for clinicians and audits of antibiotic prescription rates, and feedback to the clinicians [36]. In an example of a non-pharmacologically focused intervention, cognitive behavior therapy to treat depression was targeted using a single implementation strategy: four half-day workshops for general practitioners [16]. A few evidence-informed interventions focused on preventive care such as the management of obesity in which a single implementation strategy, a 4.5-h training program for general practitioners and nurses, was employed [17].

### Types of implementation strategies

We identified a total of 161 documented implementation strategies in the 72 articles. These strategies represented 16 of the 47 categories developed by the Cochrane EPOC group [13] and included: education material distribution (*n* = 35), audit and feedback (*n* = 32), educational meetings (*n* = 25), educational outreach visits (*n* = 22), local consensus process (*n* = 9), local opinion leaders (*n* = 6), reminders (*n* = 6), mass media (*n* = 8), patient-mediated interventions (*n* = 8), case management (*n* = 2), marketing (*n* = 1), revision of professional roles (*n* = 1), clinical multidisciplinary teams (*n* = 1), changes to the service delivery site (*n* = 1), changes in the medical record (*n* = 1), and quality monitoring mechanisms (*n* = 1). The 72 included studies in our analysis often employed more than one implementation strategy to change provider behavior: 22 studies had one strategy, 20 studies had two strategies, 16 studies had three strategies, and 14 studies had four or more. The implementation strategies targeted general practitioners (*n* = 51), other physicians and surgeons (*n* = 9), nurses or midwives (*n* = 6), and various other professionals. The majority of articles (*n* = 61; 85 %) contained implementation strategies that were directed towards a single health care provider while the 11 (15 %) remaining contained strategies that were directed towards two or more health care providers.

### Fidelity to the implementation strategies

The extent and/or quality of fidelity to the implementation strategies was documented in 51 (71 %) of the articles. Inter-rater reliability coefficients for each component of the fidelity score were as follows: adherence = 0.85, dose = 0.89, and participant responsiveness = 0.96. The mean fidelity documentation scores for the implementation strategies was 2.6 (SD = 2.25) with total scores ranging from 0 to 6. In Table 2, the mean scores, standard deviations, and the following regression results are reported for each fidelity domain. A linear regression revealed a statistically significant decline over time for the scores of adherence, *R*^2^ = 0.09, β coefficient = −0.04, *p* = 0.006; dose, *R*^2^ = 0.09, β coefficient = −0.03, *p* = 0.013; participant response, *R*^2^ = 0.14, β coefficient = −0.05, *p* = 0.001; and all domains, *R*^2^ = 0.16, β coefficient = −0.12, *p* < 0.001.

**Table 2:** Linear regression of scores of documenting fidelity to the implementation strategy by year of publication

Fidelity domain	Mean (SD)	β coefficient	*R* ^2^	*p* value
Adherence	0.93 (0.94)	−0.04	0.09	0.006
Dose	0.71 (0.88)	−0.03	0.09	0.013
Participant response	0.97 (0.93)	−0.05	0.14	0.001
All domains	2.61 (2.26)	−0.12	0.16	< 0.001

*SD* standard deviation

In 14 articles (19 %), reports of fidelity related to the implementation strategy were exemplary with scores of 2 for each of adherence, dose, and participant responsiveness [14, 15, 37–48] for a total score of 6. For example, Schaffner et al. [44] reported adherence by describing the drug educator and the physician visits to family doctors with 85 % of the visits completed. They also reported dose with 33 % of family doctors keeping the brochure, and they reported participant responsiveness using telephone evaluations with 94 % of visits reported to be friendly and 85 % reported to be useful.

## Discussion

In this scoping review, we identified the extent and quality of documentation of fidelity to the implementation strategy. Seventy-one percent of the included studies reported some details regarding the extent and/or quality of fidelity to the implementation strategies; however, details were scant for many of these studies. We did not find a single study that included a fidelity conceptual framework much less a fidelity definition. Overall, fidelity documentation scores were low in each of the three fidelity domains assessed, with a statistically significant decline in total fidelity scores over time.

Our findings of poor documentation and reporting of fidelity are consistent with findings in other scoping or systematic reviews assessing fidelity [8, 25–28]. Gearing et al. found that elements of monitoring intervention delivery (fidelity to the evidence-informed intervention) are the most commonly reported and discussed elements in the fidelity literature; while design, intervention training, and monitoring intervention receipt (dose received, client comprehension, session attendance) are the least commonly reported [26].

Reporting the fidelity to the implementation strategy enables researchers in the field of implementation science to assess the extent to which implementation success is influenced by the strategy or strategies used. In the implementation science literature, there is a wide variety of potential implementation strategies that might be utilized with a range of documented effectiveness. Reporting documentation of fidelity to the implementation strategy will facilitate selection of optimal implementation strategies, more accurate replication, and ultimately more successful transfer of evidence into practice. Without this knowledge, we lack the ability to replicate implementation strategies and ultimately translate evidence-informed strategies into practice [49, 50].

To enhance clarity around fidelity documentation of implementation strategies, we adapted an existing scoring method, proposed a revised strategy for scoring fidelity to implementation strategies, and created a checklist that can be used by other researchers. Our revisions to the Dusenbury et al. [1] criteria for capturing fidelity reflect the criteria we felt were amenable to the variety of contexts where implementation strategies could be used and represent a comprehensive assessment of both content and dose of the implementation strategy as well as the delivery process.

Although the appropriateness of adherence, the first component of the Implementation Strategy Fidelity Checklist, has been contested in the literature in relation to the value of tailoring interventions and strategies to the context of the settings where the implementation takes place [2, 51, 52], the value of *reporting* adherence is to offer clarity about what actually took place during implementation. Several studies included in this scoping review demonstrate very good reporting of adherence. For example, the authors Cockburn et al. [38] and Kiessling et al. [53] specified the implementation strategy and provided evidence of the extent to which the implementation strategy took place.

Reporting the dose of an implementation strategy enables the reader to appreciate the proportion of and extent to which the participants actually received the implementation strategy. For example, if very few received the strategy, one might question whether the strategy was effective or appropriate to participants. In this scoping review, articles reflecting very good reporting of the dose of an implementation strategy included King et al. [16] and Loeb et al. [54]. In each of these studies, researchers reported the proportion of people that received the implementation strategy. According to the definition of dose in the Table 1 checklist, attendance at an education session is sufficient to characterize dose; however, mere attendance at a session does not necessarily imply that the attendees understood the information. In the Bellg et al. BCC fidelity framework [7], the construct of “receipt” is more involved. Not only must participants attend a session, they must also display evidence of having understood the intervention and acquired the necessary competencies (i.e., knowledge/skills).

Reporting how the delivery process of an implementation strategy is received can influence future application of that strategy. It is useful to know whether the participants were actively involved with the implementation strategy and/or whether they evaluated the strategy. Examples of articles in which participant responsiveness was exceptionally well reported included Hershey et al. [15] and Soumerai et al. [46]. Both articles reported the use of 5-point Likert scales to assess physician attitudes about feedback and a newsletter in the former and receptivity and involvement in educational discussions in the latter.

Collectively, these three domains of fidelity supported our assessment of the fidelity to the implementation strategies. Successful replication of an implementation strategy will be enhanced when adherence, dose, and participant responsiveness are adequately documented and reported. Our approach to assessing the extent and quality of fidelity using a checklist is new: it is focused on implementation strategies rather than evidence-based interventions, and it is a practical and parsimonious approach that could guide researchers in the collection and reporting of data about fidelity of implementation strategies. The fidelity checklist has only been formally tested in the context of this scoping review; therefore, future research is indicated to further develop the psychometric properties of our fidelity measure.

It is somewhat surprising to observe in our sample a statistically significant decline in the quality of fidelity documentation over time given the increased use of reporting standards such as the CONSORT statement which was first published in 1996 [55, 56]. Several factors may account for the deficiency in reporting the fidelity to the implementation strategies. There has been a proliferation of interest in measuring fidelity in intervention research [57] and increased recognition of the importance of measuring implementation fidelity, but the lack of a clear conceptual definition of fidelity combined with a lack of tools to measure it likely contributes to a researcher’s inability to measure it [58] in a meaningful way. In addition, many journals do not require articles on intervention studies to report implementation fidelity [57], and authors of the reviewed studies may have limited their reporting due to journal word limits [50, 59]. During the publication period covered by our sample of articles, there was a reduction in article word limits for each of the three included journals. Authors also may have limited their reporting of fidelity because they were not attending to contextual factors that can affect the delivery of an implementation strategy. Accurate and detailed documentation of the implementation strategies may not have been prioritized.

Replication of evidence-informed interventions and application of implementation strategies will be more effective in implementation science research when all strategy components are systematically, accurately, and concisely documented. We recommend that author guidelines in journals request these details and provide a section for reporting them. Journals dedicated to publishing articles about implementation offer important forums for publishing detailed information of implementation strategies. In addition, we anticipate that an increased consistency in reporting fidelity to the implementation strategy in the implementation science literature will lead to the reporting of frameworks used to assess and report fidelity.

### Implications for future research

The findings of this review hold important implications for researchers in the field of implementation science. Fidelity information could help to advance the theoretical understanding of implementation strategies by revealing what might make one implementation strategy more effective than another or more effective in certain contexts compared with other contexts. Reporting data on fidelity could reduce the replication of unsuccessful strategies. It might also help to explain why implementation strategies such as audit and feedback have highly variable effects [60].

Given the current lack of consistency in reporting fidelity to implementation strategies, it is necessary find ways to support researchers to report these details. Researchers may want to consider a systematic approach to the reporting of fidelity. For example, in this review, we employed a scoring system for dose, adherence, and participant responsiveness, which could be a useful fidelity-reporting template for researchers. The inter-rater reliability coefficients of the scoring schema (adherence = 0.85, dose = 0.89, and participant responsiveness = 0.96) suggests that use of this scoring mechanism might be appropriate and reliable, though additional studies are needed to confirm reliability of the definitions and scoring schema.

#### Limitations

Although our search of systematic reviews in the EPOC database usefully allowed us to target articles that included implementation strategies, there were several limitations to this approach. First, searching for articles in the reference lists of systematic reviews precluded the inclusion of more recent articles in our scoping review; nevertheless, we have included articles published as recently as 2011. Second, to limit the volume of articles to be included, and thus, make the work of data extraction more feasible, our search strategy only included articles published in the three most cited journals within the systematic reviews of EPOC [20]. Our results may be subject to a publication bias because we only included articles published in three leading and high-impact medical journals; however, these journals also had the highest number of publications meeting our initial inclusion criteria. The combination of these two elements for our selection strategy allowed us to draw conclusions from high-quality, high-impact articles which are likely to influence study design, clinical practice, and patient outcomes. This methodology is consistent with other recent review articles [34, 61, 62]. Using this approach, we were able to identify the extent of documenting fidelity to a wide range of implementation strategies. Furthermore, using this search and selection process, we did observe saturation during the data extraction.

## Conclusion

In this scoping review, we identified the under-reporting of fidelity of implementation strategies in the health literature. We also developed and tested a simple checklist to assess the extent and quality of reporting fidelity of implementation strategies targeting health providers. More research is indicated to assess the definitions and scoring schema of this checklist. A fidelity framework, similar to the one used in this scoping review, will support the conduct and reporting of research activities. Careful reporting of details about fidelity of implementation strategies will make an important contribution to the field of implementation science.

## Additional files

Additional file 1:
**Definitions for populating data extraction columns.** (DOCX 13 kb) Additional file 2:
**References for the 72 included articles.** (DOCX 21 kb) Additional file 3:
**Summary of data extracted from the 72 included articles by decade.** (DOC 142 kb)

## Abbreviations

BCC: Behavior Change Consortium
EPOC: Cochrane Database of Systematic Reviews, Effective Practice/Health Systems
ERIC: Expert Recommendations for Implementing Change
