# Supplementary material for: What is the extent and quality of documentation and reporting of fidelity to implementation strategies: a scoping review
Source: Implement Sci. 2015 Sep 7;10:129. doi: 10.1186/s13012-015-0320-3 (PMC4562107; doi:10.1186/s13012-015-0320-3)
Supplement: Additional file 1: — Definitions for populating data extraction columns. (DOCX 13 kb) [file 13012_2015_320_MOESM1_ESM.docx]

Additional file 1. Definitions for Populating Data Extraction Columns

1. First Author/Year:
2. Country/Setting (e.g., primary care, intensive care unit, etc.)
3. Design / Primary Discipline(s) Targeted by Implementation Strategy (providers whose behaviours to be changed by implementation strategy)
4. Fidelity Definition (definition of fidelity noted anywhere in the article)
5. Fidelity Conceptual Framework (fidelity model, framework or theory noted anywhere in the article)
6. Implementation Strategy (as defined by EPOC)
7. Implementation Fidelity Scores (for each component of adherence, dose, and response as scored on the 3-point scale).
