# Supplementary material for: What is the extent and quality of documentation and reporting of fidelity to implementation strategies: a scoping review
Source: Implement Sci. 2015 Sep 7;10:129. doi: 10.1186/s13012-015-0320-3 (PMC4562107; doi:10.1186/s13012-015-0320-3)
Supplement: Additional file 2: — References for the 72 included articles. (DOCX 21 kb) [file 13012_2015_320_MOESM2_ESM.docx]

**1980s Decade**

1. Barnett GO, Winickoff RN, Morgan MM, Zielstorff RD: **A computer-based monitoring system for follow-up of elevated blood pressure.** *Medical care* 1983, **21:**400-409.

2. Chassin MR, McCue SM: **A randomized trial of medical quality assurance. Improving physicians' use of pelvimetry.** *Jama* 1986, **256:**1012-1016.

3. Cohen DI, Jones P, Littenberg B, Neuhauser D: **Does cost information availability reduce physician test usage? A randomized clinical trial with unexpected findings.** *Medical care* 1982, **20:**286-292.

4. Gehlbach SH, Wilkinson WE, Hammond WE, Clapp NE, Finn AL, Taylor WJ, Rodell MS: **Improving drug prescribing in a primary care practice.** *Medical care* 1984, **22:**193-201.

5. Hershey CO, Porter DK, Breslau D, Cohen DI: **Influence of simple computerized feedback on prescription charges in an ambulatory clinic. A randomized clinical trial.** *Medical care* 1986, **24:**472-481.

6. Hershey CO, Goldberg HI, Cohen DI: **The effect of computerized feedback coupled with a newsletter upon outpatient prescribing charges. A randomized controlled trial.** *Medical care* 1988, **26:**88-94.

7. Kottke TE, Brekke ML, Solberg LI, Hughes JR: **A randomized trial to increase smoking intervention by physicians. Doctors Helping Smokers, Round I.** *Jama* 1989, **261:**2101-2106.

8. Linn BS: **Continuing medical education. Impact on emergency room burn care.** *Jama* 1980, **244:**565-570.

9. McAlister NH, Covvey HD, Tong C, Lee A, Wigle ED: **Randomised controlled trial of computer assisted management of hypertension in primary care.** *British medical journal (Clinical research ed)* 1986, **293:**670-674.

10. McDonald CJ, Wilson GA, McCabe GP, Jr.: **Physician response to computer reminders.** *Jama* 1980, **244:**1579-1581.

11. McDowell I, Newell C, Rosser W: **A randomized trial of computerized reminders for blood pressure screening in primary care.** *Medical care* 1989, **27:**297-305.

12. Palmer RH, Louis TA, Hsu LN, Peterson HF, Rothrock JK, Strain R, Thompson MS, Wright EA: **A randomized controlled trial of quality assurance in sixteen ambulatory care practices.** *Medical care* 1985, **23:**751-770.

13. Schaffner W, Ray WA, Federspiel CF, Miller WO: **Improving antibiotic prescribing in office practice. A controlled trial of three educational methods.** *Jama* 1983, **250:**1728-1732.

14. Sommers LS, Sholtz R, Shepherd RM, Starkweather DB: **Physician involvement in quality assurance.** *Medical care* 1984, **22:**1115-1138.

15. Soumerai SB, Avorn J: **Economic and policy analysis of university-based drug "detailing".** *Medical care* 1986, **24:**313-331.

16. Soumerai SB, Avorn J: **Predictors of physician prescribing change in an educational experiment to improve medication use.** *Medical care* 1987, **25:**210-221.

17. Tierney WM, Hui SL, McDonald CJ: **Delayed feedback of physician performance versus immediate reminders to perform preventive care. Effects on physician compliance.** *Medical care* 1986, **24:**659-666.

18. Winickoff RN, Coltin KL, Morgan MM, Buxbaum RC, Barnett GO: **Improving physician performance through peer comparison feedback.** *Medical care* 1984, **22:**527-534.

**1990s Decade**

19. Balas EA, Boren SA, Hicks LL, Chonko AM, Stephenson K: **Effect of linking practice data to published evidence. A randomized controlled trial of clinical direct reports.** *Medical care* 1998, **36:**79-87.

20. Bird JA, McPhee SJ, Jenkins C, Fordham D: **Three strategies to promote cancer screening. How feasible is wide-scale implementation?** *Medical care* 1990, **28:**1005-1012.

21. Cockburn J, Ruth D, Silagy C, Dobbin M, Reid Y, Scollo M, Naccarella L: **Randomised trial of three approaches for marketing smoking cessation programmes to Australian general practitioners.** *BMJ (Clinical research ed)* 1992, **304:**691-694.

22. Davies G, Pyke S, Kinmonth AL: **Effect of non-attenders on the potential of a primary care programme to reduce cardiovascular risk in the population. Family Heart Study Group.** *BMJ (Clinical research ed)* 1994, **309:**1553-1556.

23. Diabetes Integrated Care Evaluation Team: **Integrated care for diabetes: clinical, psychosocial, and economic evaluation.** *BMJ (Clinical research ed)* 1994, **308:**1208-1212.

24. Dietrich AJ, O'Connor GT, Keller A, Carney PA, Levy D, Whaley FS: **Cancer: improving early detection and prevention. A community practice randomised trial.** *BMJ (Clinical research ed)* 1992, **304:**687-691.

25. Family Heart Study Group: **Randomised controlled trial evaluating cardiovascular screening and intervention in general practice: principal results of British family heart study.** *BMJ (Clinical research ed)* 1994, **308:**313-320.

26. Feder G, Griffiths C, Highton C, Eldridge S, Spence M, Southgate L: **Do clinical guidelines introduced with practice based education improve care of asthmatic and diabetic patients? A randomised controlled trial in general practices in east London.** *BMJ (Clinical research ed)* 1995, **311:**1473-1478.

27. Fender GR, Prentice A, Gorst T, Nixon RM, Duffy SW, Day NE, Smith SK: **Randomised controlled trial of educational package on management of menorrhagia in primary care: the Anglia menorrhagia education study.** *BMJ (Clinical research ed)* 1999, **318:**1246-1250.

28. Gonzales R, Steiner JF, Lum A, Barrett PH, Jr.: **Decreasing antibiotic use in ambulatory practice: impact of a multidimensional intervention on the treatment of uncomplicated acute bronchitis in adults.** *JAMA* 1999, **281:**1512-1519.

29. Imperial Cancer Research Fund OXCHECK Study Group: **Effectiveness of health checks conducted by nurses in primary care: final results of the OXCHECK study.** *BMJ (Clinical research ed)* 1995, **310:**1099-1104.

30. Kerse NM, Flicker L, Jolley D, Arroll B, Young D: **Improving the health behaviours of elderly people: randomised controlled trial of a general practice education programme.** *BMJ (Clinical research ed)* 1999, **319:**683-687.

31. Kimberlin CL, Berardo DH, Pendergast JF, McKenzie LC: **Effects of an education program for community pharmacists on detecting drug-related problems in elderly patients.** *Medical care* 1993, **31:**451-468.

32. Kinmonth AL, Woodcock A, Griffin S, Spiegal N, Campbell MJ: **Randomised controlled trial of patient centred care of diabetes in general practice: impact on current wellbeing and future disease risk. The Diabetes Care From Diagnosis Research Team.** *BMJ (Clinical research ed)* 1998, **317:**1202-1208.

33. Leviton LC, Goldenberg RL, Baker CS, Schwartz RM, Freda MC, Fish LJ, Cliver SP, Rouse DJ, Chazotte C, Merkatz IR, Raczynski JM: **Methods to encourage the use of antenatal corticosteroid therapy for fetal maturation: a randomized controlled trial.** *Jama* 1999, **281:**46-52.

34. Lomas J, Enkin M, Anderson GM, Hannah WJ, Vayda E, Singer J: **Opinion leaders vs audit and feedback to implement practice guidelines. Delivery after previous cesarean section.** *Jama* 1991, **265:**2202-2207.

35. McCartney P, Macdowall W, Thorogood M: **A randomised controlled trial of feedback to general practitioners of their prophylactic aspirin prescribing.** *BMJ (Clinical research ed)* 1997, **315:**35-36.

36. Modell M, Wonke B, Anionwu E, Khan M, Tai SS, Lloyd M, Modell B: **A multidisciplinary approach for improving services in primary care: randomised controlled trial of screening for haemoglobin disorders.** *BMJ (Clinical research ed)* 1998, **317:**788-791.

37. Nattinger AB, Hoffmann RG, Howell-Pelz A, Goodwin JS: **Effect of Nancy Reagan's mastectomy on choice of surgery for breast cancer by US women.** *Jama* 1998, **279:**762-766.

38. O'Connell DL, Henry D, Tomlins R: **Randomised controlled trial of effect of feedback on general practitioners' prescribing in Australia.** *BMJ (Clinical research ed)* 1999, **318:**507-511.

39. Premaratne UN, Sterne JA, Marks GB, Webb JR, Azima H, Burney PG: **Clustered randomised trial of an intervention to improve the management of asthma: Greenwich asthma study.** *BMJ (Clinical research ed)* 1999, **318:**1251-1255.

40. Schectman JM, Kanwal NK, Schroth WS, Elinsky EG: **The effect of an education and feedback intervention on group-model and network-model health maintenance organization physician prescribing behavior.** *Medical care* 1995, **33:**139-144.

41. Soumerai SB, McLaughlin TJ, Gurwitz JH, Guadagnoli E, Hauptman PJ, Borbas C, Morris N, McLaughlin B, Gao X, Willison DJ, et al: **Effect of local medical opinion leaders on quality of care for acute myocardial infarction: a randomized controlled trial.** *Jama* 1998, **279:**1358-1363.

42. Soumerai SB, Salem-Schatz S, Avorn J, Casteris CS, Ross-Degnan D, Popovsky MA: **A controlled trial of educational outreach to improve blood transfusion practice.** *Jama* 1993, **270:**961-966.

43. Wyatt JC, Paterson-Brown S, Johanson R, Altman DG, Bradburn MJ, Fisk NM: **Randomised trial of educational visits to enhance use of systematic reviews in 25 obstetric units.** *BMJ (Clinical research ed)* 1998, **317:**1041-1046.

**2000s Decade**

44. Austin PC, Mamdani MM, Tu K, Jaakkimainen L: **Prescriptions for estrogen replacement therapy in Ontario before and after publication of the Women's Health Initiative Study.** *Jama* 2003, **289:**3241-3242.

45. Austin PC, Mamdani MM, Tu K, Zwarenstein M: **Changes in prescribing patterns following publication of the ALLHAT trial.** *Jama* 2004, **291:**44-45.

46. Beck CA, Richard H, Tu JV, Pilote L: **Administrative Data Feedback for Effective Cardiac Treatment: AFFECT, a cluster randomized trial.** *Jama* 2005, **294:**309-317.

47. Berner ES, Baker CS, Funkhouser E, Heudebert GR, Allison JJ, Fargason CA, Jr., Li Q, Person SD, Kiefe CI: **Do local opinion leaders augment hospital quality improvement efforts? A randomized trial to promote adherence to unstable angina guidelines.** *Medical care* 2003, **41:**420-431.

48. Cheater FM, Baker R, Reddish S, Spiers N, Wailoo A, Gillies C, Robertson N, Cawood C: **Cluster randomized controlled trial of the effectiveness of audit and feedback and educational outreach on improving nursing practice and patient outcomes.** *Medical care* 2006, **44:**542-551.

49. Eccles M, McColl E, Steen N, Rousseau N, Grimshaw J, Parkin D, Purves I: **Effect of computerised evidence based guidelines on management of asthma and angina in adults in primary care: cluster randomised controlled trial.** *BMJ (Clinical research ed)* 2002, **325:**941.

50. Ferguson TB, Jr., Peterson ED, Coombs LP, Eiken MC, Carey ML, Grover FL, DeLong ER, Society of Thoracic S, the National Cardiac D: **Use of continuous quality improvement to increase use of process measures in patients undergoing coronary artery bypass graft surgery: a randomized controlled trial.** *Jama* 2003, **290:**49-56.

51. Figueiras A, Sastre I, Tato F, Rodriguez C, Lado E, Caamano F, Gestal-Otero JJ: **One-to-one versus group sessions to improve prescription in primary care: a pragmatic randomized controlled trial.** *Medical care* 2001, **39:**158-167.

52. Figueiras A, Herdeiro MT, Polonia J, Gestal-Otero JJ: **An educational intervention to improve physician reporting of adverse drug reactions: a cluster-randomized controlled trial.** *Jama* 2006, **296:**1086-1093.

53. Flottorp S, Oxman AD, Havelsrud K, Treweek S, Herrin J: **Cluster randomised controlled trial of tailored interventions to improve the management of urinary tract infections in women and sore throat.** *BMJ (Clinical research ed)* 2002, **325:**367.

54. Gill CJ, Phiri-Mazala G, Guerina NG, Kasimba J, Mulenga C, MacLeod WB, Waitolo N, Knapp AB, Mirochnick M, Mazimba A, et al: **Effect of training traditional birth attendants on neonatal mortality (Lufwanyama Neonatal Survival Project): randomised controlled study.** *BMJ (Clinical research ed)* 2011, **342:**d346.

55. Hersh AL, Stefanick ML, Stafford RS: **National use of postmenopausal hormone therapy: annual trends and response to recent evidence.** *Jama* 2004, **291:**47-53.

56. Kiefe CI, Allison JJ, Williams OD, Person SD, Weaver MT, Weissman NW: **Improving quality improvement using achievable benchmarks for physician feedback: a randomized controlled trial.** *Jama* 2001, **285:**2871-2879.

57. Kiessling A, Henriksson P: **Efficacy of case method learning in general practice for secondary prevention in patients with coronary artery disease: randomised controlled study.** *BMJ (Clinical research ed)* 2002, **325:**877-880.

58. King M, Davidson O, Taylor F, Haines A, Sharp D, Turner R: **Effectiveness of teaching general practitioners skills in brief cognitive behaviour therapy to treat patients with depression: randomised controlled trial.** *BMJ* 2002, **324:**947-950.

59. Loeb M, Brazil K, Lohfeld L, McGeer A, Simor A, Stevenson K, Zoutman D, Smith S, Liu X, Walter SD: **Effect of a multifaceted intervention on number of antimicrobial prescriptions for suspected urinary tract infections in residents of nursing homes: cluster randomised controlled trial.** *BMJ (Clinical research ed)* 2005, **331:**669.

60. Majumdar SR, Almasi EA, Stafford RS: **Promotion and prescribing of hormone therapy after report of harm by the Women's Health Initiative.** *Jama* 2004, **292:**1983-1988.

61. Mason J, Freemantle N, Browning G: **Impact of effective health care bulletin on treatment of persistent glue ear in children: time series analysis.** *BMJ (Clinical research ed)* 2001, **323:**1096-1097.

62. Moher M, Yudkin P, Wright L, Turner R, Fuller A, Schofield T, Mant D: **Cluster randomised controlled trial to compare three methods of promoting secondary prevention of coronary heart disease in primary care.** *BMJ (Clinical research ed)* 2001, **322:**1338.

63. Moore H, Summerbell CD, Greenwood DC, Tovey P, Griffiths J, Henderson M, Hesketh K, Woolgar S, Adamson AJ: **Improving management of obesity in primary care: cluster randomised trial.** *BMJ (Clinical research ed)* 2003, **327:**1085.

64. Morrison J, Carroll L, Twaddle S, Cameron I, Grimshaw J, Leyland A, Baillie H, Watt G: **Pragmatic randomised controlled trial to evaluate guidelines for the management of infertility across the primary care-secondary care interface.** *BMJ (Clinical research ed)* 2001, **322:**1282-1284.

65. Pandey P, Sehgal AR, Riboud M, Levine D, Goyal M: **Informing resource-poor populations and the delivery of entitled health and social services in rural India: a cluster randomized controlled trial.** *Jama* 2007, **298:**1867-1875.

66. Perz JF, Craig AS, Coffey CS, Jorgensen DM, Mitchel E, Hall S, Schaffner W, Griffin MR: **Changes in antibiotic prescribing for children after a community-wide campaign.** *Jama* 2002, **287:**3103-3109.

67. Sehgal AR, Leon JB, Siminoff LA, Singer ME, Bunosky LM, Cebul RD: **Improving the quality of hemodialysis treatment: a community-based randomized controlled trial to overcome patient-specific barriers.** *Jama* 2002, **287:**1961-1967.

68. Stafford RS, Furberg CD, Finkelstein SN, Cockburn IM, Alehegn T, Ma J: **Impact of clinical trial results on national trends in alpha-blocker prescribing, 1996-2002.** *Jama* 2004, **291:**54-62.

69. Tu JV, Donovan LR, Lee DS, Wang JT, Austin PC, Alter DA, Ko DT: **Effectiveness of public report cards for improving the quality of cardiac care: the EFFECT study: a randomized trial.** *Jama* 2009, **302:**2330-2337.

70. van Eijk ME, Avorn J, Porsius AJ, de Boer A: **Reducing prescribing of highly anticholinergic antidepressants for elderly people: randomised trial of group versus individual academic detailing.** *BMJ (Clinical research ed)* 2001, **322:**654-657.

71. Verstappen WH, van der Weijden T, Sijbrandij J, Smeele I, Hermsen J, Grimshaw J, Grol RP: **Effect of a practice-based strategy on test ordering performance of primary care physicians: a randomized trial.** *Jama* 2003, **289:**2407-2412.

72. Welschen I, Kuyvenhoven MM, Hoes AW, Verheij TJ: **Effectiveness of a multiple intervention to reduce antibiotic prescribing for respiratory tract symptoms in primary care: randomised controlled trial.** *BMJ (Clinical research ed)* 2004, **329:**431.
