# Supplementary material for: What is the extent and quality of documentation and reporting of fidelity to implementation strategies: a scoping review
Source: Implement Sci. 2015 Sep 7;10:129. doi: 10.1186/s13012-015-0320-3 (PMC4562107; doi:10.1186/s13012-015-0320-3)
Supplement: Additional file 3: — Summary of data extracted from the 72 included articles by decade. (DOC 142 kb) [file 13012_2015_320_MOESM3_ESM.doc]

**Additional file 3: Summary of Included Articles by Decade**

1980s Decade

| **Author /**  **Year** | **Country/ Setting** | **Design /Primary Discipline Targeted by the Implementation Strategy** | **Evidence-Based Innovation** | **Implementation Strategy^a^** | **Implementation Strategy Fidelity Scores**  **(Domain Range: 0-2)** |
| --- | --- | --- | --- | --- | --- |
| Barnett 1983 | US; General practice | RCT; physicians | Follow-up of newly identified elevated diastolic blood pressure (BP) | Professional intervention^8^ | Adherence- 0  Dose- 0  Participant Response- 0 |
| Chassin 1986 | US | RCT; physicians | Reduce inappropriate use of x-ray pelvimetry | Professional intervention^1, 3, 4, 7^ | Adherence- 2  Dose- 0  Participant Response- 2 |
| Cohen 1982 | US;  Hospital | RCT; physicians | Expenditures for health care/diagnostic tests and x-rays (physician ordering patterns) | Professional interventions^4, 7^ | Adherence- 0  Dose- 0  Participant Response- 1 |
| Gelhbach 1984 | US;  family practice medical centre | RCT; physicians and medical residents | Prescribing patterns | Professional intervention^7^ | -Adherence- 0  Dose- 0  Participant Response- 2 |
| Hershey 1986 | US;  Ambulatory Clinics | RCT; medical residents | Decrease cost of Prescriptions | Professional Intervention^7^ | Adherence- 2  Dose- 0  Participant Response- 2 |
| Hershey 1988 | US;  Medical outpatient | RCT; physicians | Prescribing patterns | Professional Intervention^1, 7^ | Adherence- 2  Dose- 2  Participant Response- 2 |
| Kottke 1989 | US | RCT; physicians | Promoting smoking cessation | Professional intervention^1, 2, 6^ | Adherence- 2  Dose- 1  Participant Response- 0 |
| Linn 1980 | US | RCT; ER physicians | Improve process of emergency room burn care | Professional intervention^1, 2, 7^ | Adherence- 1  Dose- 2  Participant Response- 2 |
| McAlister 1986 | Canada; Family practices | RCT; physicians | Community Hypertension Management Project | Professional Intervention^7^ | Adherence- 2  Dose- 2  Participant Response- 1 |
| McDonald  1980 | US;  Hospitals | Observational cohort study;  physicians | Clinical reminders about events that need corrective action | Professional intervention^8^ | Adherence- 2  Dose- 2  Participant Response- 2 |
| McDowell 1989 | Canada;  Primary care | RCT; physician | Blood Pressure Screening | Professional intervention^8^ | Adherence- 2  Dose- 0  Participant Response- 1 |
| Palmer 1985 | US; Primary care and pediatric care | RCT; general practitioners | Quality Assurance | Professional interventions^1, 2, 7^ | Adherence- 2  Dose- 2  Participant Response- 2 |
| Schaffner  1983 | USA  Physician offices | RCT; physicians | Antibiotic prescribing practices | Professional interventions^1, 4^ | Adherence- 2  Dose- 2  Participant Response- 2 |
| Sommers 1984 | US; Hospital | Controlled trial; physicians | Evidence-based criteria low hemoglobin levels treatment | Professional interventions^3, 7, 8^ | Adherence- 2  Dose- 2  Participant Response- 2 |
| Soumerai 1986 | US; office-based medical practice | RCT; physician | Reduction of inappropriate drug prescribing | Professional interventions^1, 4^ | Adherence- 1  Dose- 2  Participant Response- 1 |
| Soumerai 1987 | US;  office-based medical practice | RCT; physician | Reduction of inappropriate drug prescribing and improvement in prescribing | Professional interventions^1, 4, 7^ | Adherence- 2  Dose- 2  Participant Response- 2 |
| Tierney 1986  Yes | US; General practice | RCT; general physicians | Preventative care protocols | Professional interventions^7, 8^ | Adherence- 0  Dose- 0  Participant Response- 2 |
| Winickoff 1984 | US; General practice | RCT and pre-test post-test; physicians | Colorectal screening | Professional interventions^1, 2, 3, 7^ | Adherence- 2  Dose- 2  Participant Response- 2 |

^a^ Implementation strategy categories defined by EPOC [27]

^1^ Distribution of educational material

^2^ Educational meetings

^3^ Local consensus process

^4^ Educational outreach visits

^6^ Patient-mediated interventions

^7^ Audit and feedback

^8^ Reminders

1990s Decade

| **Author /**  **Year** | **Country/ Setting** | **Design /Primary Discipline Targeted by the Implementation Strategy** | **Evidence-Based Innovation** | **Implementation Strategy^a^** | **Implementation Strategy Fidelity Scores**  **(Domain Range: 0-2)** |
| --- | --- | --- | --- | --- | --- |
| Balas 1998 | US | RCT; physicians | Selection between two types of dialysis (CAPD/CCPD) | Professional interventions ^1, 7^ | Adherence- 2  Dose- 0  Participant Response- 0 |
| Bird 1990 | US; Internal Medicine | RCT; residents/Medical Trainees | Cancer-screening Tests | Professional interventions ^2, 6, 7, 8^ | Adherence- 2  Dose- 2  Participant Response- 2 |
| Cockburn  1992 | Australia; Primary care | RCT; general practitioners | Smoking Cessation kit to be used for patients by provider | Professional intervention ^1, 4^ | Adherence- 2  Dose- 2  Participant Response- 2 |
| Davies 1994 | UK; General practices | RCT; general practice nurses  - Nurse follow-up of non-attenders to the Family Heart Study Programme | Follow-up program to reduce cardiovascular risk | Professional intervention ^2^ | Adherence- 0  Dose- 0  Participant Response- 0 |
| Diabetes Integrated Care Evaluation Team  1994 | UK;  Hospital diabetic clinic and 3 general practices | Pragmatic randomised trial; general practitioners | Integrated care for diabetes in general practice | Professional intervention ^6, 8^ | Adherence- 1  Dose- 0  Participant Response- 0 |
| Dietrich  1992 | US; ambulatory care | RCT; physicians | Early cancer detection and preventative services | Professional intervention ^2, 29, 33b^ | Adherence- 2  Dose- 2  Participant Response- 2 |
| Family Heart Study Group/  Wood 1994 | UK; General Practices | RCT; Nurses | Nurse-led programme using family centred approach with follow-up | Professional intervention ^2^ | Adherence- 0  Dose- 0  Participant Response- 0 |
| Feder  1995 | UK;  General practice | RCT; general practitioners | Clinical guidelines for asthma and diabetes | Professional intervention ^1, 3, 7, 8^ | Adherence- 1  Dose- 0  Participant Response- 2 |
| Fender 1999 | UK | RCT; primary care doctors | Referral and treatment for menorrhagia | Professional interventions ^1, 4^ | Adherence- 0  Dose- 0  Participant Response- 0 |
| Gonzales  1999 | US;  Primary care practices | Prospective non-randomized controlled trial;  physicians & nurses | Antibiotic prescriptions for uncomplicated acute bronchitis | Professional intervention ^1, 2, 7, 10^ | Adherence- 2  Dose- 0  Participant Response- 0 |
| Imperial Cancer Research Fund  1995 | UK; General practices | Descriptive; general practice nurses | OXCHECK; Nurse-administered screenings in primary care | Professional intervention ^2^ | Adherence- 0  Dose- 0  Participant Response- 0 |
| Kerse  1999 | Australia; General practices | RCT; general practitioners | Health promotion in general practice | Professional Intervention ^1^ | Adherence- 0  Dose- 0  Participant Response- 1 |
| Kimberlin  1993 | US; community | Experimental design; pharmacists | Assessing presenting patients and drug therapy choices | Professional  Interventions ^1, 2, 7^ | Adherence- 0  Dose- 0  Participant Response- 0 |
| Kinmonth  1998 | England; general practice | Pragmatic RCT; general practitioners and nurses | Improving Diabetic care | Professional interventions ^1, 4^ | Adherence- 1  Dose- 1  Participant Response- 2 |
| Leviton  1999 | US;  Hospitals | RCT  Physicians | Antenatal corticosteriods | Professional interventions ^2, 3, 5, 7, 8^ | Adherence- 2  Dose- 1  Participant Response- 1 |
| Lomas 1991 | Canada | Cluster RCT; physicians | Guideline-based management of women with previous caesarean section | Professional interventions ^2, 3, 5, 7^ | Adherence- 2  Dose- 2  Participant Response- 2 |
| McCartney  1997 | UK; general practices | RCT; general practitioners | Prescribing practices for aspirin for ischemic heart disease | Professional intervention ^4, 7^ | Adherence- 0  Dose- 0  Participant Response- 0 |
| Modell 1998 | UK;  General practices | Practice-based RCT;  physicians and nurses | Screening for carriers of haemoglobin disorders | Professional interventions ^1, 4, 8, 30^ | Adherence- 2  Dose- 1  Participant Response- 2 |
| Nattinger 1998 | US | Observational cohort study; hospital physicians | Breast cancer surgery | Professional intervention ^10^ | Adherence- 0  Dose- 0  Participant Response- 0 |
| O’Connell 1999 | Australia | RCT; general practitioners | Prescribing practices for 5 main drug groups | Professional intervention ^1, 7^ | Adherence- 0  Dose- 0  Participant Response- 0 |
| Premaratne 1999 | UK | RCT; practice nurses | Asthma management | Professional intervention ^2, 4, 5^ | Adherence- 2  Dose- 0  Participant Response- 0 |
| Shectman 1995 | US; primary care practices | RCT; primary care physicians | Prescribing of H2-blockers | Professional interventions ^1, 7^ | Adherence- 0  Dose- 0  Participant Response- 0 |
| Soumerai 1998 | US | RCT; hospital physicians | Acute MI guideline implementation | Professional Interventions ^1, 2, 3, 5, 7, 40^ | Adherence- 0  Dose- 0  Participant Response- 2 |
| Soumerai  1993 | USA;  Hospital | RCT  physicians | Appropriateness of blood product utilization (transfusion) | Professional intervention ^1, 4^ | Adherence- 0  Dose- 1  Participant Response- 0 |
| Wyatt 1998 | UK | RCT; physicians and midwives | Using evidence in practice | Professional interventions ^4^ | Adherence- 2  Dose- 0  Participant Response- 0 |

^a^ Implementation strategy categories defined by EPOC [27]

^1^ Distribution of educational material

^2^ Educational meetings

^3^ Local consensus process

^4^ Educational outreach visits

^5^ local opinion leaders

^6^ Patient-mediated interventions

^7^ Audit and feedback

^8^ Reminders

^10^ Mass media

^22^ Formulary

^30^ Clinical multidisciplinary teams

^33b^ Case-management

^40^ Changes in medical record systems

2000s Decade

| **Author /**  **Year** | **Country/ Setting** | **Design /Primary Discipline Targeted by the Implementation Strategy** | **Evidence-Based Innovation** | **Implementation Strategy^a^** | **Implementation Strategy Fidelity Scores**  **(Domain Range: 0-2)** |
| --- | --- | --- | --- | --- | --- |
| Austin 2003 | Canada | Observational Cohort Study; physicians | Estrogen replacement therapy (ERT) | Professional intervention ^10^ | Adherence- 0  Dose- 0  Participant Response- 0 |
| Austin 2004 | Canada | Observational Cohort Study; physicians | Incident use of antihypertensive agents | Professional intervention ^10^ | Adherence- 0  Dose- 0  Participant Response- 0 |
| Beck  2005 | Canada | Cluster RCT; multi-disciplinary | Cardiac treatment | Professional intervention ^1, 7^ | Adherence- 2  Dose- 2  Participant Response- 2 |
| Berner 2003 | US; Inpatient hospital | Group RCT; physicians | Unstable angina (UA) guidelines | Professional interventions ^2, 5, 8^  Structural interventions ^39, 42^ | Adherence- 0  Dose- 0  Participant Response- 0 |
| Cheater 2006 | UK;  Family practice | Cluster RCT; community nurses | Assessment and management of urinary incontinence | Professional interventions ^1, 4, 7^ | Adherence- 0  Dose- 2  Participant Response- 2 |
| Eccles  2002 | UK; Primary care | Cluster RCT; general practitioners | Evidence-based guideline recommender practice for asthma and angina | Professional intervention ^1, 2, 33b^ | Adherence- 0  Dose- 0  Participant Response- 2 |
| Ferguson 2003 | US | Cluster RCT; physicians/surgeons | Coronary artery by-pass graft surgery | Professional intervention ^1, 5, 7^ | Adherence- 2  Dose- 0  Participant Response- 1 |
| Figueiras 2001 | Spain; Family practice | Pragmatic RCT; physicians | Prescribing patterns of nonsteroidal anti-inflammatory drugs (NSAIDS) | Professional interventions ^1, 2, 4, 8^ | Adherence- 2  Dose- 2  Participant Response- 0 |
| Figueieras 2006  Yes | Portugal | Cluster RCT; physicians | Adverse drug reaction (ADR) reporting of physicians | Professional intervention ^4, 7, 8^ | Adherence- 0  Dose- 1  Participant Response- 0 |
| Flottorp 2002 | Norway; general practices | Cluster RCT; general practitioners | Implementation of guidelines for urinary tract infection and sore throat in women | Professional interventions ^1, 2, 6, 8^ | Adherence- 0  Dose- 0  Participant Response- 0 |
| Gill  2011 | Zambia | Prospective, cluster randomized and controlled effectiveness study  Traditional birth attendants | Providing attendants with skills targeting birth asphyxia neonatal hypothermia, and sepsis | Professional interventions ^2^ | Adherence- 1  Dose- 1  Participant Response- 0 |
| Hersh  2004 | US; Primary care | Database study; physicians | Hormone therapy | Professional intervention ^10^ | Adherence- 0  Dose- 0  Participant Response- 0 |
| Kiefe  2001 | US;  Outpatient care | RCT;  physician | Diabetes care, influenza vaccine, foot examination, 3 blood tests | Professional Intervention ^2, 7^ | Adherence- 0  Dose- 0  Participant Response- 2 |
| Kiessling 2002 | Sweden; General practices | Prospective Control Trial; General practitioners | Local guidelines for secondary prevention of coronary artery disease | Professional intervention ^1, 2, 3, 4, 5^ | Adherence- 2  Dose- 0  Participant Response- 1 |
| King  2002 | UK;  General practices | Parallel group, cluster RCT; general Practitioners | Cognitive behaviour therapy | Professional intervention ^4^ | Adherence- 1  Dose- 1  Participant Response- 2 |
| Loeb et al  2005 | Canada and US;  Nursing homes | Cluster RCT; nurses and physicians | Antimicrobial prescriptions for suspected urinary tract infections (changing prescription patterns) | Professional interventions ^1, 2, 4^ | Adherence- 0  Dose- 0  Participant Response- 0 |
| Majumdar 2004 | US | Prospective longitudinal data; pharmacists | Use of hormone replacement therapy | Professional intervention ^10^ | Adherence- 0  Dose- 0  Participant Response- 0 |
| Mason et al  2001 | UK;  Hospital | Time series analysis; surgeons | Treatment of persistent glue ear; surgery rates | Professional intervention ^1^ | Adherence- 0  Dose- 0  Participant Response- 0 |
| Moher 2001 | UK | RCT; general practitioners and nurse | Secondary prevention of coronary artery disease | Professional interventions ^7, 8^ | Adherence- 0  Dose- 0  Participant Response- 2 |
| Moore 2003 | UK; General practices | Cluster randomized trial; general practitioners and general practice nurses | Obesity Management | Professional ^1^ | Adherence- 2  Dose- 0  Participant Response- 2 |
| Morrison 2001 | UK; General practices  and hospitals | Cluster RCT; general practitioners and hospitals accepting referrals for infertility | Clinical guidelines for infertility treatment and referral across primary and secondary care settings | Professional interventions ^1, 2, 4^ | Adherence- 1  Dose- 2  Participant Response- 1 |
| Pandey 2007 | India | Cluster randomized RCT; nurse midwives | Delivery of health and social services | Professional interventions ^1, 6^ | Adherence- 2  Dose- 2  Participant Response- 2 |
| Perz  2002 | US | Pre-post quasi-experimental; healthcare practitioners (physicians, providers, hospital staff) | Reduce inappropriate antibiotic prescribing | Professional interventions ^1, 5, 6, 10^ | Adherence- 0  Dose- 0  Participant Response- 0 |
| Sehgal 2002 | US | RCT; nephrologists | Improve adequacy of hemodialysis | Professional interventions ^1, 4, 6, 7^ | Adherence- 1  Dose- 1  Participant Response- 2 |
| Stafford 2004 | US | Observational Cohort study; physicians | Prescribing of beta-blockers | Professional intervention ^9, 10^ | Adherence- 0  Dose- 0  Participant Response- 0 |
| Tu 2009 | Canada | Population-based, Cluster RCT; hospital-level | Improve healthcare processes and outcomes for cardiac care quality process | Professional intervention ^7, 10^ | Adherence- 0  Dose- 1  Participant Response- 1 |
| Van Eijk 2001 | Netherlands | RCT; general practitioners and pharmacists | Prescribing patterns for anti-depressants for older patients | Professional Interventions ^4^ | Adherence- 2  Dose- 2  Participant Response- 2 |
| Verstappen 2003 | Netherlands | Cluster RCT; primary care physicians | Improving diagnostic test ordering guidelines | Professional interventions ^1, 2, 7^ | Adherence- 0  Dose- 1  Participant Response- 0 |
| Welschen 2004 | Netherlands | RCT; general practitioners and pharmacists | Prescribing patterns for antibiotics | Professional interventions ^2, 3, 7^ | Adherence- 0  Dose- 0  Participant Response- 0 |

^a^ Implementation strategy categories defined by EPOC [27]

^1^ Distribution of educational material

^2^ Educational meetings

^3^ Local consensus process

^4^ Educational outreach visits

^5^ local opinion leaders

^6^ Patient-mediated interventions

^7^ Audit and feedback

^8^ Reminders

^9^ Marketing

^10^ Mass media

^33b^ Case-management

^39^ Changes to the setting/site of service delivery

^42^ Presence and organization of quality monitoring mechanisms
